# Investigation of the Quasi-Binary Phase Diagram FLiNaK-NdF_3_

**DOI:** 10.3390/ma14216428

**Published:** 2021-10-26

**Authors:** Peter Mushnikov, Olga Tkacheva, Vladimir Voronin, Vladimir Shishkin, Yuriy Zaikov

**Affiliations:** Institute of High Temperature Electrochemistry of the Ural Branch of the Russian Academy of Sciences, 620990 Ekaterinburg, Russia; O.Tkacheva@ihte.uran.ru (O.T.); V.Voronin@ihte.uran.ru (V.V.); V.Shishkin@ihte.uran.ru (V.S.); Zaikov@ihte.uran.ru (Y.Z.)

**Keywords:** molten salt reactor, solubility, FLiNaK, neodymium trifluoride

## Abstract

The NdF_3_ solubility in molten eutectic FLiNaK, which is a conceivable medium for a molten salt reactor (MSR), was determined by the quasi-binary phase diagram FLiNaK-NdF_3_. The eutectic mixture FLiNaK was prepared by direct melting of components LiF, NaF and KF·HF. The acidic anhydrous salt (KF·HF) was used instead of the hygroscopic KF. The NdF_3_ was sintered by hydrofluorination of Nd_2_O_3_. The oxygen impurity in the prepared eutectic FLiNaK, determined by an oxygen analyzer LECO OH836, was 0.036 wt.%, whereas the NdF_3_ contained 0.04 wt.% of oxygen. A part of the FLiNaK-NdF_3_ quasi-binary phase diagram was obtained using two thermal analysis techniques: differential thermal analysis (DTA) and differential scanning calorimetry (DSC). The FLiNaK-NdF_3_ phase diagram in the region of 0–30 mol.% NdF_3_ contains one eutectic at 2 mol.% NdF_3_ and 450 °C and two peritectic points: 8 mol.% NdF_3_ at 500 °C and 22 mol.% NdF_3_ at 575 °C. The region of the FLiNaK-NdF_3_ phase diagram below the liquidus line is rather complicated due to the complex structure of the multicomponent system in its molten state, as in its solid state. The NdF_3_ solubility in FLiNaK is about 5 mol.% at 490 °C and 29 mol.% at 700 °C; this means that the process of the MA transmutation in the MSR can be carried out in molten FLiNaK with a content of actinides as high as 15–20 mol.% in the temperature range of 550–650 °C.

## 1. Introduction

Reprocessing spent nuclear fuel (SNF) is a high-tech process with the purpose of minimizing the radiation hazard of this fuel, ensuring the safe disposal of unused components, and facilitating the release of useful substances (primarily uranium and plutonium). At the same time, to ensure economic efficiency and improve environmental safety, the issue of the minor actinides (MA) arises [1,2,3,4,5]. The MA, which are long-lived radioactive isotopes of americium, curium and neptunium, all transuranic chemical elements, are primary contributors to the high radioactivity of the waste remaining after SNF processing. The extraction of MA from the SNF and their subsequent “burning” reduces the amount of highly active solidified waste intended for deep disposal and allows multiple returns to the nuclear fuel cycle of uranium and plutonium. It is possible to efficiently process MA by transmutation, that is, by “burning” into the molten salt nuclear reactors (MSR). Such facilities have several advantages, including increased safety, since due to their technological features, severe accidents cannot occur [6]. In addition, such reactors do not require the manufacturing of traditional fuel cells.

The MSR design and its operating mode substantially depend on the actinides content in the fuel. The most important criterion for choosing a salt solvent is the solubility of actinide fluorides in molten salt media. Some molten fluoride mixtures (LiF-NaF-BeF_2_, NaF-ZrF_4_, NaF-ZrF_4_, LiF-BeF_2_, LiF-NaF-KF) have been considered as candidate salt solvents [7,8,9,10,11]. One of the most promising solvents is the eutectic FLiNaK, which has a melting point of 454 °C, and, therefore, it can provide an operating temperature in the range of 550–650 °C [12,13].

However, the solubility values of most actinide fluorides in molten FLiNaK are not known. It should be noted that an experiment related to the actinide fluorides’ solubility in molten salt requires quite complex equipment, duration time and material costs [14]. Studies of actinide analogues—lanthanides, which are similar in physicochemical, thermodynamic, and crystallographic properties—are much more efficient to carry out. It is known [14,15] that CeF_3_ and NdF_3_ are analogues of PuF_3_ and AmF_3_, and UF_4_ can serve as a simulator of NpF_4_.

The solubility of rare earth fluorides in the molten eutectic FLiNaK was investigated by several researchers [14,16,17,18]. Usually, studies were carried out by isothermal saturation using complex design cells and various ways of data acquisition (including visual). It is also important to provide an inert atmosphere in the cells. The measurements were performed in the range of high concentrations of REM fluoride; nevertheless, the interval between measurement points was large enough. As a result, the data obtained by different researchers often do not coincide [14,18].

The more correct approach for studying solubility is to obtain phase diagrams that give a clear idea of the homogeneity area of a salt system. Phase diagrams of multicomponent fluoride systems can be developed, on the one hand, by experimental methods of thermal analysis, and, on the other hand, by modeling and computer simulation using thermodynamical and thermochemical data [19].

Usually, binary or ternary systems of alkali and REM fluorides have been studied [20,21,22,23,24]. Thus, Berkani and Gaune-Escard [20] performed experiments on the modeling and thermodynamic computation of the binaries NdF_3_-MF (M = Li, Na, K). The authors [21] compared the results of the thermodynamic modeling and available experimental data [25] for the systems LiF-LnF_3_ (Ln = La-Sm). The phase diagrams of such binaries represented diagrams with a single eutectic without the formation of new compounds. The physicochemical properties of the (LiF-NaF)_eut_-LaF_3_ system with the LaF_3_ content up to 25 mol.% were reported in paper [22]. The phase equilibria were measured by thermal analyses (TA) and differential scanning calorimetry. The (LiF-NaF)_eut_-LaF_3_ phase diagram below the liquidus line is quite complex: several crystallizing compounds can be identified. The authors [23] found that in the LiF-NaF-LaF_3_ system there are two ternary invariant points, eutectic and peritectic. Experimental investigation using TA and thermodynamic modeling of the LiF-NdF_3_-DyF_3_ was accomplished by Abbasalizadeh et al. [24]. The LiF-NdF_3_ system has a simple eutectic and no solid solubility in its cubic and hexagonal phases. The optimized eutectic composition for this system is 22 mol.% NdF_3_ at 734 °C. Fedorov [26,27] presented the phase diagrams of the MF-NdF_3_ (M = K, Na) binary systems in a wide range of the NdF_3_ concentration, according to which each phase diagram has one eutectic and three peritectic points. Although the phase diagrams of binary and some ternary systems are available, the quasi-binary diagrams FLiNaK-(REM)F_3_ have not been attained yet.

The purpose of this work was to develop a quasi-binary phase diagram FLiNaK-NdF_3_ in the concentration range of NdF_3_ 0–30 mol.% using thermal analysis techniques and to determine the NdF_3_ solubility in molten eutectic FLiNaK.

## 2. Materials and Methods

### 2.1. FLiNaK Preparation

The eutectic mixture FLiNaK of the composition (mol.%) 46.5 LiF—11.5 NaF—42 KF was prepared by direct melting of components. The following individual salts were used: lithium fluoride LiF (VECTON, RF), wherein the mass fraction of LiF was 99.0%; sodium fluoride NaF (GRANCHIM), wherein the mass fraction of NaF was 99.0%; acid potassium fluoride KF·HF (GRANCHIM), wherein the mass fraction of KF·HF was 99–101%.

A distinctive feature of the technique for obtaining the eutectic FLiNaK is the use of an acidic anhydrous salt KF·HF (KHF_2_) instead of the hygroscopic KF. The melting point of KHF_2_ is 238.7 °C, and the boiling point is in the range of 400–500 °C. The decomposition reaction of KHF_2_ proceeds already at temperatures of 300–400 °C.

The direct melting of components using the acid salt KF·HF has several advantages, such as the simplicity of the technological scheme, and the low concentration of impurities due to the natural fluorination of the melt upon decomposition of the KF·HF during slow heating.

**Figure 1:**
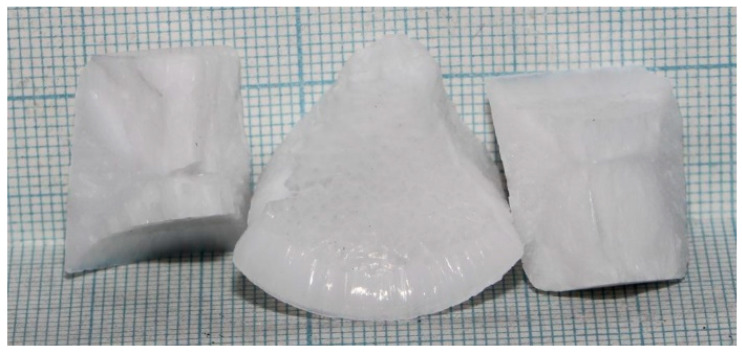
Photo of prepared eutectic FLiNaK.

A glassy-carbon crucible (400 mL) with a weighted amount of components was heated to 750 °C with a rate of about 2.5 °C/min for 5–6 h. The melt was sustained for about 2 h at 750 °C. Then, the melt was cooled and transferred to a glovebox with a controlled atmosphere (humidity not more than 2 ppm, oxygen content 2–9 ppm). Visual observation showed that the salt had a pure white color without accessory inclusions on the surface (Figure 1).

An elemental chemical analysis of the main components and impurities in the prepared eutectic FLiNaK was carried out by an inductively coupled plasma optical emission spectrometer (ICP-OES) iCAP 6300 Duo (Thermo Scientific, Waltham, MA, USA).

The experimentally determined concentrations (C_exp_) of the main components LiF, NaF, and KF in the eutectic mixture FLiNaK are summarized in Table 1. The deviation of experimental concentrations from theoretical values (C_theor_.) is represented as the C_exp_/C_theor_. ratio. It follows from the Table 1 that the concentrations of components are close to the theoretical values. The maximum deviation of the LiF, NaF and KF concentrations from the required eutectic composition does not exceed 5%.

The oxygen content was determined using an oxygen analyzer LECO OH836. The average oxygen impurity in the prepared FLiNaK eutectic was found to be 0.036 ± 0.005 wt%.

**Figure 2:**
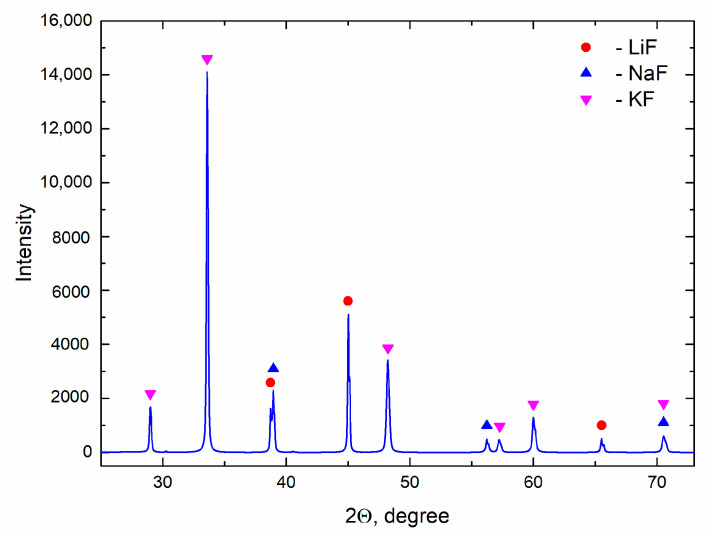
The X-ray diffraction pattern of prepared FLiNaK.

The FLiNaK sample of frozen salt was subjected to XRD analysis using Rigaku MiniFlex 600 (Rigaku, Tokyo, Japan). The diffraction pattern is given in Figure 2. Three phases with cubic structures, LiF (a = 4.03019(2) Å), NaF (a = 4.62912(3) Å) and KF (a = 5.34189(3) Å), were found by the Rietveld full-profile analysis using the FulProf program.

### 2.2. Neodymium Trifluoride Preparation

Neodymium trifluoride was prepared by hydrofluorination of Nd_2_O_3_. The neodimiun oxide (99.995%) was dissolved in dilute hydrochloric acid according to the reaction:

Nd_2_O_3_ + 6HCl → 2NdCl_3_ + 3H_2_O. (1)

The chloride solution was transferred to a glassy carbon bowl and evaporated. Then, the concentrated hydrofluoric acid was added in a stoichiometric amount to convert NdCl_3_ to NdF_3_.

The obtained suspension was evaporated on a hot plate to a stiff consistency, and hydrofluoric acid was added again. The operations of acid addition and evaporation were repeated three times in order to completely remove chlorine ions. After drying, sintered pieces of NdF_3_ were crushed in an agate mortar, loaded into a glassy carbon container, and annealed with slow heating to 800 °C under vacuum in order to remove residual HF.

**Figure 3:**
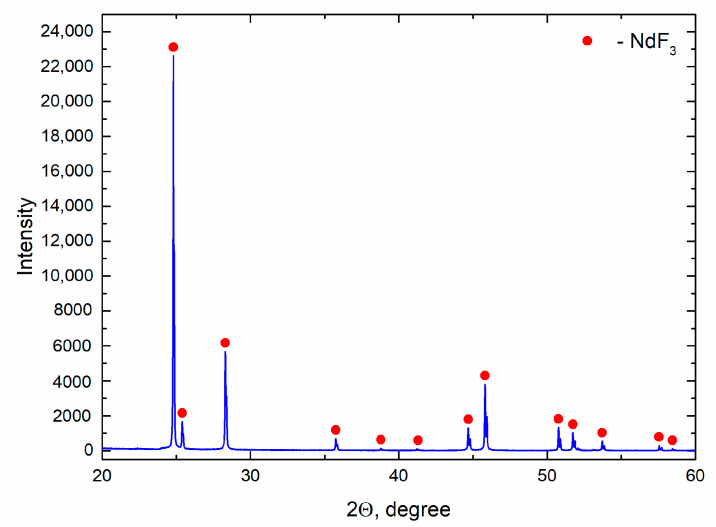
The X-ray diffraction pattern of prepared NdF_3_.

The results of the XRD analysis are presented in Figure 3. The Rietveld full-profile analysis confirmed the presence of a single hexagonal phase with lattice parameters a = 7.03148(5) Å, c = 7.19883(7) Å.

Analysis of the oxygen content using oxygen analyzer LECO OH836 indicated that its content in the prepared NdF_3_ does not exceed 0.04 wt%.

### 2.3. Differential Thermal Analysis

The differential thermal analysis (DTA) consisted of measuring the sample temperature or the temperature difference between the sample and the reference versus time during cooling. All experiments were carried out in a glove box with a controlled inert atmosphere of argon, in which the moisture and oxygen content did not exceed 1 ppm.

A glassy carbon crucible filled with FLiNaK (15–30 g) was placed in a nickel container covered with a nickel cap, which had holes for thermocouple and tube for adding and sampling. The assembly was mounted into a furnace installed in the glove box.

The melt temperature was measured with a thermocouple (type K) placed in a nickel case. In order to check the thermocouple readings, the liquidus temperature of two compositions, LiF-CaF_2_ (80.5–19.5 mol.%, T_liq_ = 769 °C) and LiF-NaF-KF (46.5–11.5–42.0 mol.%, T_liq_ = 454 °C), was measured before each experiment. The cooling rate was 5 °C/min.

**Figure 4:**
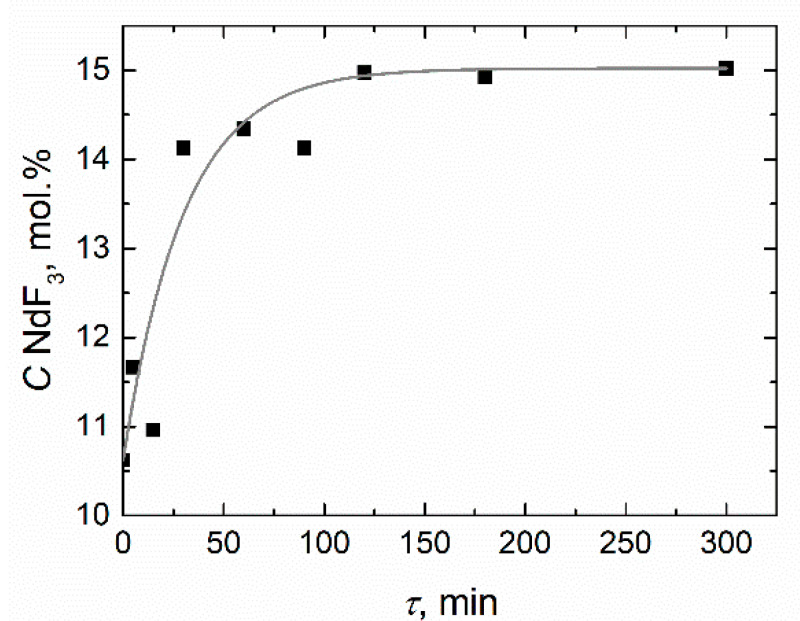
The dissolution dynamics of the NdF_3_ (5 mol.%) addition in the molten FLiNaK–NdF_3_(10 mol.%) at 700 °C.

A pre-weighed amount of NdF_3_ was discharged into the molten salt through a nickel tube. Time of the complete dissolution of the NdF_3_ additive was determined according to the Nd content in the samples analyzed by ICP. Samples were taken from the central part of the crucible carefully so as not to mix the melt. The sampler was a small spoon made of nickel, allowing about 50 mg of the melt to be scooped up. The dissolution dynamics of the NdF_3_ addition (5 mol.%) in the molten FLiNaK containing 10 mol.% NdF_3_ at 700 °C are presented in Figure 4. The time of complete dissolution of the additive was about 2 h. However, the molten mixture was sustained at constant temperature for 4 h before each measurement.

**Figure 5:**
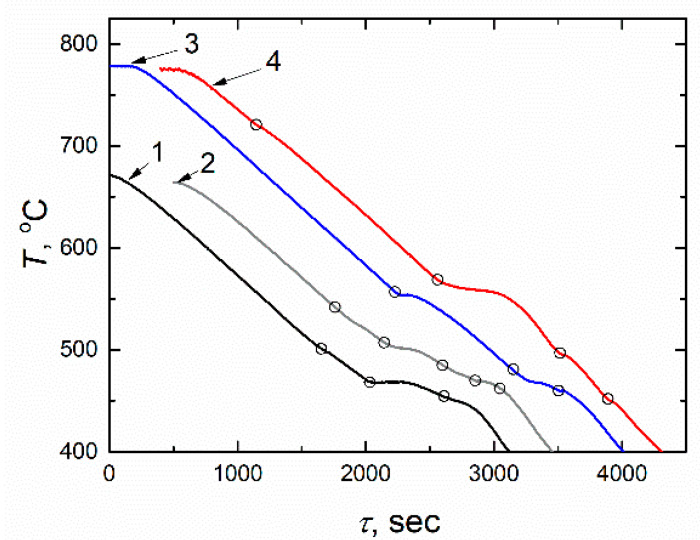
Temperature versus time during cooling of FLiNaK with different NdF_3_ contents (mol.%): 1—6, 2—15, 3—20, 4—30.

The temperature change versus time during cooling obtained in the FLiNaK with the NdF_3_ additives in the amount of 6, 15, 20, and 30 mol.% is given in Figure 5. All cooling curves have several inflection points corresponding to phase transitions. So, for example, the liquidus temperature is 502.6 °C for the FLiNaK + 6 mol.% NdF_3_ composition and is 722.9 °C for the FLiNaK + 30 mol.% NdF_3_ composition.

**Figure 6:**
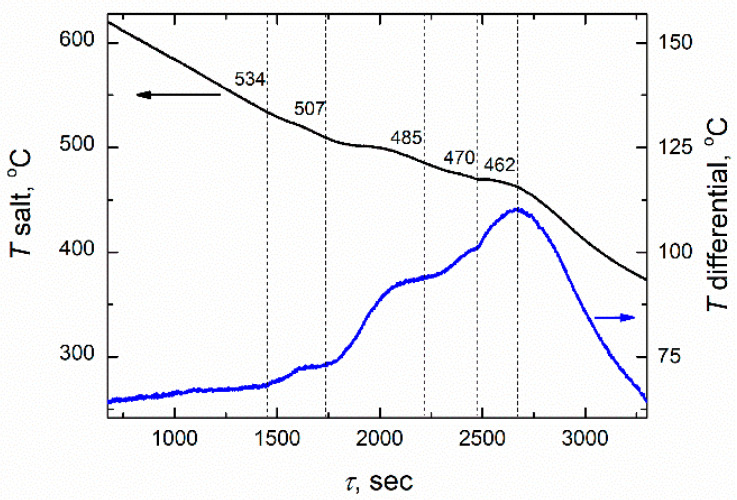
Cooling and differential curves obtained in the system FLiNaK + 15 mol.% NdF_3_.

To determine more accurately the crystallization temperature, the DTA were used. Using this technique, it is possible to record even small changes in the temperature, since the recording thermocouples from the sample and the reference are connected towards each other. Thus, by comparing the differential and cooling curves, it is achievable to determine not only the liquidus temperatures, but also the temperatures corresponding to subsequent solid phase transformations. For instance, the cooling and differential curves obtained in the system FLiNaK + 15 mol.% NdF_3_ are given in Figure 6. In addition to the liquidus (534 °C) and solidus temperature (462 °C), three temperature points corresponding to the phase transitions between different crystal modifications at 507, 485 and 470 °C were found.

### 2.4. Differential Scanning Calorimetry

Some compositions of the FLiNaK-NdF_3_ system were analyzed by differential scanning calorimetry (DSC) using a Netzsch STF 449 F3 Jupiter (Netzsch, Selb, Deutschland). The measurements were carried out in graphite crucibles in an argon atmosphere. The heating rate was 10 °C/min.

Samples for the DSC analysis were prepared in advance. About 10 mg of the studied salt mixture was placed in a nickel crucible, the diameter of which corresponded to the diameter of the DSC graphite crucible. The nickel crucible with salt was heated in a furnace located in a glovebox with an inert atmosphere. After melting and quenching, the sample had the shape of a drop with a flat bottom, which provided good contact with the bottom of the DSC crucible. The graphite crucible for the DSC analysis was used instead of the platinum crucible, because it was found that the molten salt spread along the walls of platinum crucible and the contact area of the salt with the bottom of the crucible changed.

**Figure 7:**
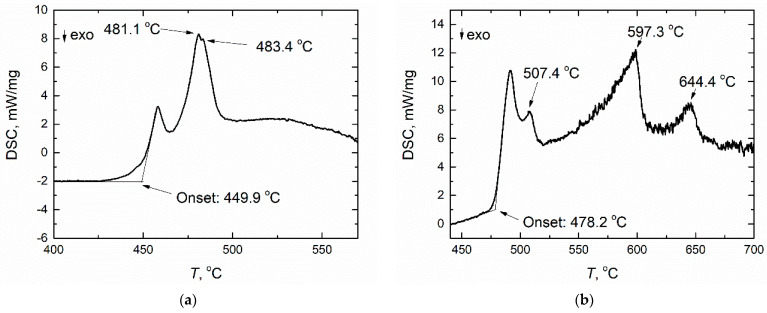
The DSC heating curves obtained (**a**) in the FLiNaK + 6 mol% NdF_3_; (**b**) in the FLiNaK + 26 mol% NdF_3_ (**b**).

The DSC curves obtained in FLiNaK containing 6 and 26 mol.% NdF_3_ are given in Figure 6. Several endothermic peaks were determined in the curve corresponding to the FLiNaK + 26 mol% NdF_3_ composition (Figure 7), which confirms the complexity of the FLiNaK-NdF_3_ diagram.

## 3. Results and Discussion

**Figure 8:**
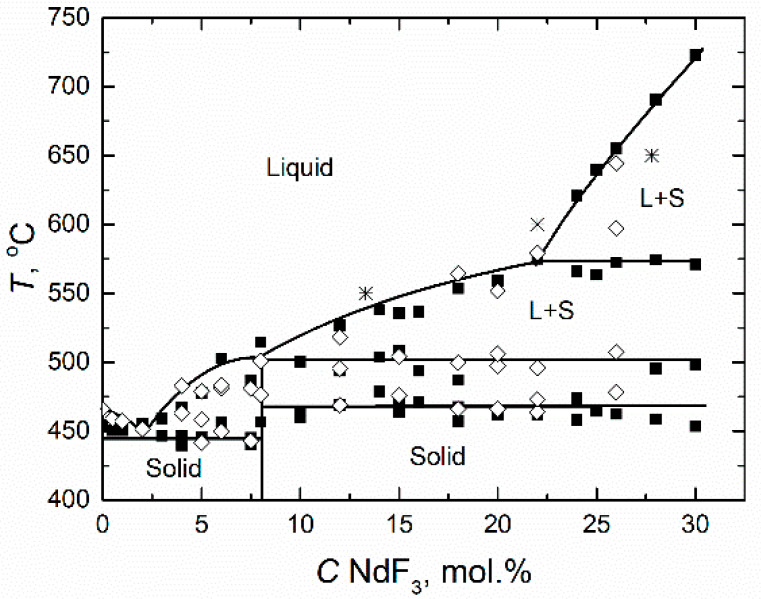
Phase diagram of the FLiNaK-NdF_3_ system: ■—TA by cooling curves; ◊—DSC; *—data [14].

The FLiNaK-NdF_3_ phase diagram in the concentration range from 0 to 30 mol.% NdF_3_ is presented in Figure 8. It was found that the phase diagram FLiNaK-NdF_3_ had one eutectic at 2 mol.% NdF_3_ and 450 °C, and two peritectic points: 8 mol.% NdF_3_ at 500 °C and 22 mol.% NdF_3_ at 575 °C. The values of the NdF_3_ solubility in the eutectic FLiNaK obtained by DTA and DSC are in good agreement. The NdF_3_ solubility increased from 5 to 25 mol.% in the temperature range of 490–640 °C. The data obtained by authors [14] using the isothermal saturation method is plotted in the same diagram. The results correlate well.

The liquidus temperature diminishes with the first NdF_3_ additions. At NdF_3_ concentrations ranging from 0 to 3 mol%, the liquidus temperature of the FLiNaK-NdF_3_ mixture is lower than the melting point of FLiNaK, and the temperature range, at which both solid and liquid phases exist in the system, does not exceed 15 °C. A further increase in the NdF_3_ content leads to a growth in the liquidus temperature up to 500 °C, while the solidus temperature does not change and is 445 °C. At higher NdF_3_ concentrations, the crystallization temperature of the melt is 470 °C.

The region of the FLiNaK-NdF_3_ melt homogeneity within the temperature range of 600–700 °C is quite wide. This means that the process of the MA transmutation in the MSR can be carried out in the molten FLiNaK with the content of actinides as high as 15–20 mol.%.

On the other hand, the region of the FLiNaK-NdF_3_ phase diagram below the liquidus line is rather complicated: the lines corresponding to the phase crystallization at 503, 468 and 445 °C have a sufficiently large scatter of points. This is due to the complex structure of the multicomponent system in its molten state, as in its solid state. In the alkali fluorides melts, the NdF_3_ was found to exist in the form of complex ions NdF_6_^3−^ and NdF_4_^−^ [28]. Moreover, the NdF_6_^3−^ decomposes readily with increasing temperature. If LiF does not form compounds with NdF_3_, then NaF and KF can form intermediate compounds of the compositions (M = Na, K) MNdF_4_, M_2_NdF_5_, M_3_NdF_6_, MNd_2_F_7_, MNd_3_F_10_ [23]. The MF-NdF_3_ binary systems contain phases of various compositions, the relevant data on which are very different [25,27]. In addition, all lanthanide trifluorides with an orthorhombic structure transform to a hexagonal crystal structure upon heating [24].

**Figure 9:**
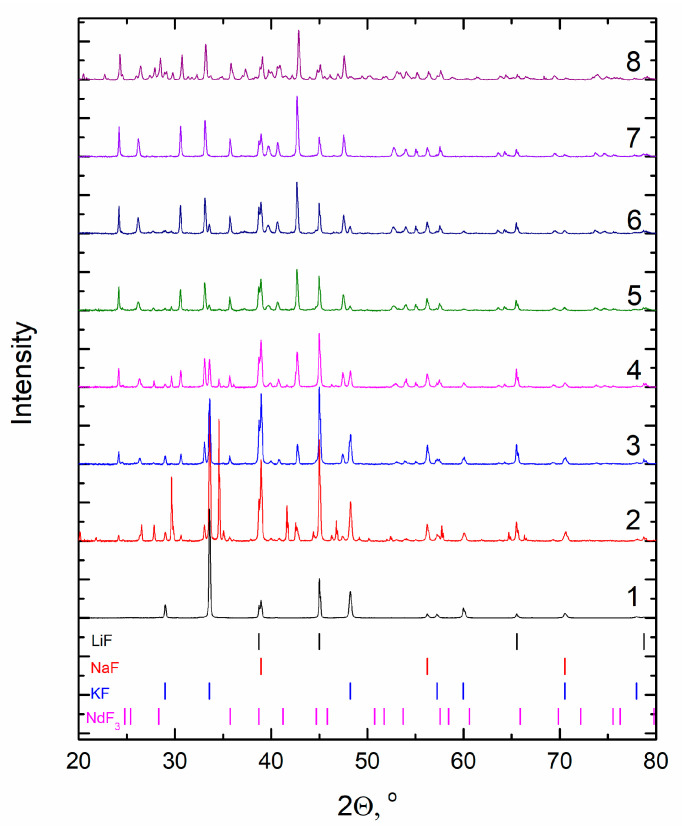
XRD patterns of (1) FLiNaK; (2) FLiNaK + 0.5 mol.% NdF_3_; (3) FLiNaK + 1.0 mol.% NdF_3_; (4) FLiNaK + 2.0 mol.% NdF_3_; (5) FLiNaK + 3.0 mol.% NdF_3_; (6) FLiNaK + 4.0 mol.% NdF_3_; (7) FLiNaK + 5.0 mol.% NdF_3_; (8) FLiNaK + 7.5 mol.% NdF_3_.

The phase composition of the FLiNaK-NdF_3_ mixtures after cooling to room temperature was analyzed by the XRD. The patterns are presented in Figure 9.

**Figure 10:**
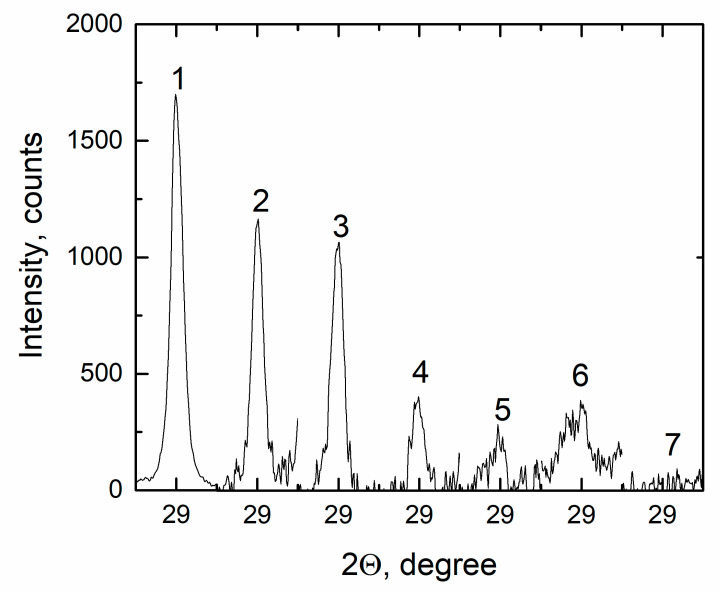
Intensity of the reflection (111) of the KF phase (1) FLiNaK; (2) FLiNaK + 0.5 mol.% NdF_3_; (3) FLiNaK + 1.0 mol.% NdF_3_; (4) FLiNaK + 2.0 mol.% NdF_3_; (5) FLiNaK + 3.0 mol.% NdF_3_; (6) FLiNaK + 4.0 mol.% NdF_3_; (7) FLiNaK + 5.0 mol.% NdF_3_.

The pattern (1) corresponds to the initial eutectic FLiNaK, the analysis of which is given in Figure 2. In Figure 9, significant changes in the diffraction pattern are visible even with minor additions of NdF_3_, e.g., pattern (2) of the FLiNaK + 0.5 mol.% NdF_3_, in which, along with reflexes corresponding to the initial phases, other reflexes were manifested. Consequently, in the process of heating and cooling, one or more additional phases are observed to form. With an increase in the NdF_3_ concentration in the system FLiNaK-NdF_3_, a continuous transformation of the set of reflections, their angular position and intensity occurs. Upon that, the set of reflections belonging to the initial eutectic is retained at least up to the 4.0 mol.% NdF_3_ (6). In the XRD patterns of compositions with a higher concentration of NdF_3_, no reflections of the initial KF phase are found; its complete dissolution has come. This is clearly seen in the example of the dependence of the intensity of the reflection (111) of the KF phase (Figure 10).

**Figure 11:**
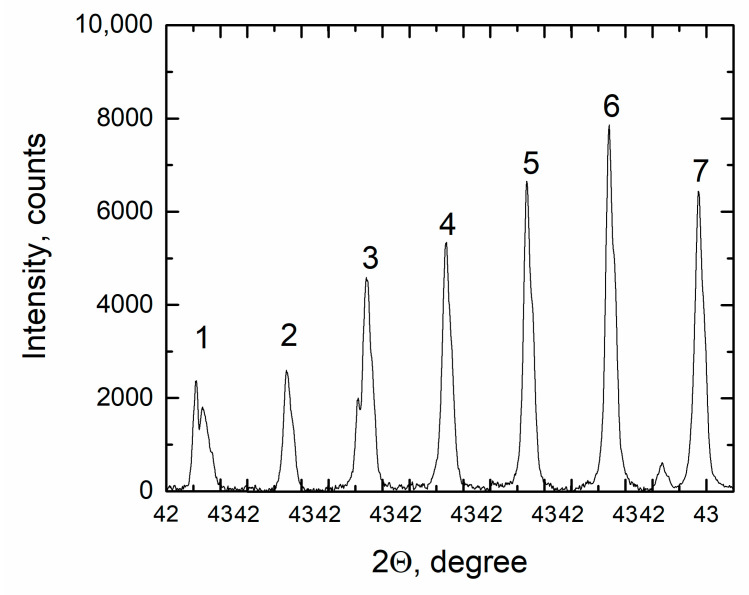
Intensity of the reflection orthorhombic phase for (1) FLiNaK + 0.5 mol.% NdF_3_; (2) FLiNaK + 1.0 mol.% NdF_3_; (3) FLiNaK + 2.0 mol.% NdF_3_; (4) FLiNaK + 3.0 mol.% NdF_3_; (5) FLiNaK + 4.0 mol.% NdF_3_; (6) FLiNaK + 5.0 mol.% NdF_3_; (7) FLiNaK + 7.5 mol.% NdF_3_.

A decrease in the peak intensity corresponding to the NaF and KF phases with an increase in the NdF_3_ concentration occurs unevenly and to a much lesser extent. The XRD pattern of the frozen sample FLiNaK + 5 mol% NdF_3_ contains reflections of the initial phases LiF and NaF, excluding which the remaining reflections can be described by an orthorhombic lattice with the following parameters: a = 13.6285(1) Å, b = 7.65102(7) Å, c = 3.83085(5) Å. This phase is present in all samples containing NdF_3_, and its content continuously rises to an NdF_3_ content of 5 mol%, which is confirmed by an increase in the main reflection of this phase in the angular region of 42–43 degrees (Figure 11). It was not possible to identify this phase using the PDF-2 2019 database; most likely, it is a complex compound containing four cations (K, Na, Li, Nd), which has not been previously synthesized as an individual substance.

## 4. Conclusions

The quasi-binary phase diagram FLiNaK-NdF_3_ was obtained by two thermal analysis techniques: DTA and DSC. It has one eutectic and two peritectic points in the region of NdF_3_ concentration 0–30 mol.%. The values of the liquidus temperature obtained by both techniques are in a good agreement. The NdF_3_ solubility in the molten eutectic FLiNaK was determined in wide temperature and concentration ranges. Its values are 5 mol.% at 490 °C and 29 mol.% at 700 °C, which testifies to the existence of a wide homogeneous region at temperatures of 550–650 °C and the sufficiency of the temperature margin for the implementation of thermohydraulic operating conditions for the MSR based on FLiNaK. Taking into account the closeness of the crystallographic and physicochemical properties of neodymium and americium trifluorides, it can be assumed that the AmF_3_ solubility in the molten FLiNaK will also be sufficiently high in the indicated temperature range.

## Figures and Tables

**Table 1:** Theoretical and experimental content (mol.%) of LiF, NaF and KF in prepared eutectic FLiNaK.

Component	C_theor_.	C_exp_	C_exp_/C_theor_.
LiF	46.5	47.67	1.03
NaF	11.5	11.50	1.0
KF	42.0	40.13	0.96

## Data Availability

The data presented in this study are available on request from the corresponding author.
